# Nutritional Risk and 30‐Day In‐Hospital Mortality in Adults With Sepsis and Septic Shock: A Retrospective Cohort Study

**DOI:** 10.1155/jonm/3010166

**Published:** 2026-09-09

**Authors:** Jeeyoung Park, JiYeon Choi, Suk Jeong Lee, Youn-Jung Son

**Affiliations:** ^1^ Department of Nursing, Seoul National University Hospital, Seoul, South Korea, snuh.org; ^2^ College of Nursing, Mo-Im Kim Nursing Research Institute, Yonsei University, Seoul, South Korea, yonsei.ac.kr; ^3^ Red Cross College of Nursing, Chung-Ang University, Seoul, South Korea, cau.ac.kr

**Keywords:** adults, intensive care units, nutritional risk, sepsis, septic shock

## Abstract

**Background:**

Despite the improvements in the management of sepsis, mortality remains high. Understanding the significance of nutritional status on mortality is vital for advancing therapeutic interventions. The aim of this study was to evaluate the relationship between patients’ nutritional status during their stay in intensive care unit (ICU) and 30‐day in‐hospital mortality among adults admitted with sepsis and septic shock.

**Methods:**

A retrospective cohort study was carried out at a single tertiary institution in South Korea between January 2015 and December 2024. The inclusion of a sample of 355 ICU patients suffering from sepsis or septic shock who survived the first seven days of the study and received enteral nutrition was a part of the study, comprising 247 survivors and 108 nonsurvivors. Data included baseline characteristics, as well as clinical and nutritional parameters collected throughout the ICU stay.

**Results:**

A 30‐day in‐hospital mortality rate of 30.4% was observed among patients with sepsis and septic shock. Each 10% increase in protein adequacy during ICU Days 4–7 was linked to a 9% lower hazard of 30‐day in‐hospital mortality (*p* = 0.043). A high nutritional risk, as defined by a modified Nutrition Risk in the Critically Ill (mNUTRIC) score, was independently associated with a 68% higher hazard of 30‐day in‐hospital mortality (*p* = 0.041). The use of continuous renal replacement therapy (*p* = 0.003), serum lactate ≥ 2 mmol/L on Day 7 (*p* < 0.001), and mean arterial pressure < 65 mmHg on Day 7 (*p* = 0.047) were also linked to a higher risk of 30‐day in‐hospital mortality.

**Conclusions:**

The nutritional status of patients with sepsis and septic shock during their stay in the ICU was found to be a significant factor in determining 30‐day in‐hospital mortality. Integrating nutritional and physiological indicators could help identify patients at greater risk of mortality and inform continued nutritional monitoring in the intensive care setting.

**Implication for Nursing Management:**

Our findings highlight the clinical importance of monitoring protein adequacy throughout the ICU care of patients with sepsis and septic shock. Nurse managers should promote standardized nutritional screening, systematic intake monitoring, and multidisciplinary collaboration to support timely, individualized nutrition care.

## 1. Introduction

Despite substantial advances in critical care and antimicrobial therapies, sepsis still remains linked to persistently high mortality worldwide [1]. In critically ill individuals with sepsis and septic shock, the reported mortality rates range from approximately 30–50%, with notable variation across healthcare systems and geographic regions [2]. In South Korea, the annual number of sepsis‐related deaths has risen to approximately 7,000, representing nearly a threefold increase over the past decade [3, 4]. In particular, the likelihood of 30‐day in‐hospital mortality following admission to the Intensive Care Unit (ICU) is influenced by both baseline patient characteristics at admission and dynamic clinical factors that evolve throughout the ICU course [5]. Established contributors to mortality include multiple comorbidities, immunosuppressive status, low body weight, presence of septic shock, reduced mean arterial pressure (MAP), elevated lactate levels, and gastrointestinal dysfunction [6, 7]. Despite standardized sepsis management, including hemodynamic support, lactate‐guided resuscitation, and organ support when indicated [1], mortality among ICU patients with sepsis remains substantial.

Accordingly, there has been an increasing focus on nutritional status as a potentially modifiable determinant of ICU outcomes [8, 9]. Individuals suffering from sepsis and septic shock exhibit profound metabolic alterations characterized by acute hypercatabolism and impaired gastrointestinal perfusion, necessitating individualized nutritional strategies [10]. Inadequate integration of clinical status and nutritional support may exacerbate metabolic dysregulation and organ dysfunction during the acute phase, potentially prolonging mechanical ventilation and increasing mortality risk [11]. But there is limited evidence about the most appropriate timing, modality, and quantity of nutritional support in cases of sepsis, and the current international guidelines offer inconsistent recommendations [12, 13].

According to the Society of Critical Care Medicine (SCCM), early enteral nutrition (EN) should begin within 72 h of ICU admission when feasible [1]. In contrast, the European Society for Clinical Nutrition and Metabolism (ESPEN) advises deferring early EN in critically ill patients requiring vasopressor support and instead advocates a gradual introduction of EN following stabilization [14]. These divergent recommendations present challenges in clinical decision‐making, particularly given concerns that early feeding in the setting of impaired systemic perfusion may exacerbate intestinal hypoperfusion [15]. Emerging evidence suggests that increased nutritional provision during the late acute phase might be connected with improved clinical outcomes [16]. Nonetheless, the definition of this phase and the corresponding nutritional targets remain inconsistent across studies. Nutritional status reflects the dynamic interplay between nutritional requirements, intake, and metabolic processes [17], and its accurate assessment therefore requires integration of nutritional risk, clinical parameters, and feasible delivery strategies [18].

The nutritional requirements of individuals with sepsis and septic shock evolve dynamically in response to changes in clinical status. Although previous studies have linked the mNUTRIC score, nutritional intake, and clinical indicators with mortality, these factors have often been examined separately or primarily in relation to baseline nutritional risk [19–21]. Consequently, there is limited evidence regarding the connection between phase‐specific energy and protein adequacy and mortality, when nutritional risk, routes of nutrition delivery, and dynamic indicators of clinical instability are considered simultaneously.

The aim of this study was to evaluate the association of nutritional status with the mortality rate within 30 days of hospitalization among adult patients suffering from sepsis or septic shock. These patients had survived the initial 7 days of their ICU admission and had received EN. Specifically, this study examined phase‐specific energy and protein adequacy, nutritional risk, routes of nutrition delivery, and dynamic indicators of clinical instability in relation to 30‐day in‐hospital mortality.

## 2. Methods

### 2.1. Study Design, Setting, and Study Sample

**FIGURE 1:**
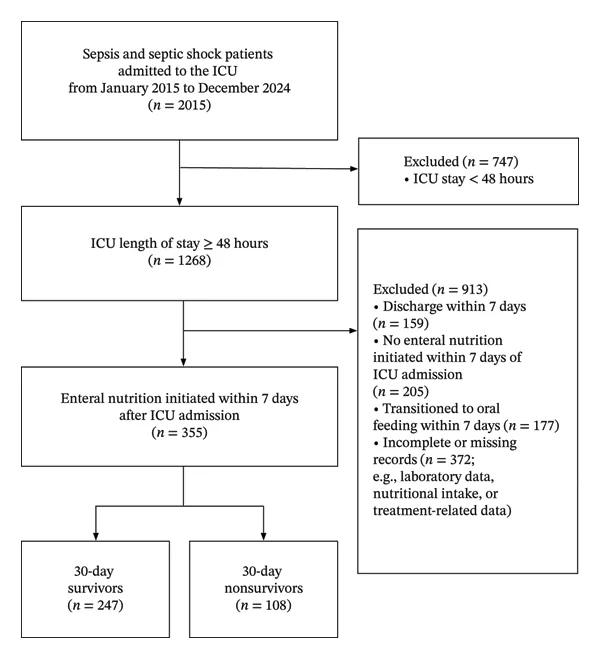
Flowchart of the patients included in this study.

This retrospective cohort study was performed at a tertiary hospital in Seoul, based on electronic medical record data collected between January 2015 and December 2024. The study population comprised adult patients aged 19 or over who were admitted to the ICU with a main diagnosis of sepsis or septic shock. Eligibility criteria included an ICU stay of at least 48 h and receipt of EN within 7 days of admission. Patients who died within the first 7 days, were discharged within 7 days, transitioned to oral nutrition, or did not receive EN were excluded. ICU patients suffering from sepsis or septic shock were classified into survival (*n* = 247) and mortality (*n* = 108) groups based on 30‐day in‐hospital mortality following ICU admission (Figure 1).

### 2.2. Measures

Data were collected on general characteristics, ICU admission characteristics, nutritional risk, nutritional parameters, nutrition delivery routes, and clinical variables. General characteristics included age, sex, alcohol consumption, smoking status, and major underlying medical conditions. At ICU admission, the following variables were assessed: body mass index (BMI), severity indices including the Sequential Organ Failure Assessment (SOFA) and Acute Physiology and Chronic Health Evaluation II (APACHE II), type of sepsis, type of ICU, MAP, and blood lactate levels [1]. MAP and blood lactate were evaluated using measurements obtained on the day of admission (Day 1) and on Day 7. A MAP < 65 mmHg was defined as hemodynamic instability [1]. Hyperlactatemia was delineated as a blood lactate level of 2 mmol/L or greater [22]. Clinical characteristics during hospitalization included mechanical ventilation, continuous renal replacement therapy (CRRT), vasopressor use, MAP on Day 7, blood lactate on Day 7, gastrointestinal complications [23], and refeeding syndrome [24], along with the length of stay in the ICU.

In the current study, nutritional status was assessed by mNUTRIC score, nutritional parameters, and nutrition delivery route. First, the mNUTRIC score was used to assess nutritional risk at ICU admission. This is a validated tool for patients with critical illness [25]. An mNUTRIC score ≥ 6, derived from ROC analysis, was used as an exploratory threshold for high nutritional risk. Nutritional parameters included total caloric intake, protein intake, and the degree to which these met predefined target requirements. As nutritional parameters, nutritional intake was evaluated from Day 1 (ICU admission) through Day 7. The timing of nutritional support was categorized as either early (Days 1–3) or late (Days 4–7) according to prior studies and clinical guidelines [14, 21]. Total caloric intake comprised contributions from EN, parenteral nutrition (PN), propofol preparations, and glucose solutions. The nutrient adequacy ratio was expressed as a percentage. This was calculated by dividing the mean nutritional intake by the target requirement. The definition of target requirements as 25 kcal/kg/day for energy and 1.3 g/kg/day for protein was based on the American Society for Parenteral and Enteral Nutrition (ASPEN) and ESPEN guidelines [14, 26]. For patients with a BMI ≥ 30 kg/m^2^, target requirements were calculated using adjusted body weight (AjBW) to decrease the risk of overfeeding. AjBW was calculated as the ideal body weight (IBW) + 0.25 × (actual body weight − IBW), with IBW estimated using the Devine formula: For males, this is 50 + 0.9 × (height in cm − 152), and for females, it is 45.5 + 0.9 × (height in cm − 152) [27]. Nutrition delivery routes were categorized as EN alone or a combination of EN and PN. The definition of PN is the administration of total PN formulations for nutritional support [14].

### 2.3. Outcome Variable

The main clinical outcome was 30‐day in‐hospital mortality after ICU admission, a commonly used short‐term outcome measure in critical care research [28].

### 2.4. Data Collection

After we obtained authorization to reach the electronic medical records, we extracted data with the help of standardized case report forms. Data sources included nursing admission assessments, physician documentation, nursing notes, clinical observation records, order entries, laboratory results, and consultation records.

### 2.5. Data Analysis

We performed the statistical analyses with IBM SPSS Statistics Version 31.0 (IBM Corp., Armonk, NY, USA). For all the tests, we used two‐tailed tests and a level of significance of *p* < 0.05. General and clinical characteristics, nutritional status, and 30‐day in‐hospital mortality were summarized using descriptive statistics. The Mann–Whitney U test and chi‐square (*χ* ^2^) test were used to compare survival groups. An exploratory threshold for nutritional risk was obtained using receiver operating characteristic (ROC) curve analysis. A comparison was made of 30‐day survival rates between low‐ and high‐risk groups using Kaplan–Meier survival analysis. Cox proportional hazards regression was applied to investigate the factors related to 30‐day in‐hospital mortality. Patients who were discharged alive within 30 days were considered survivors, and their follow‐up time was calculated up to the date of discharge. The selection of covariates was carried out on the basis of clinical relevance, as supported by prior literature, essential demographic factors, and univariate associations (*p* < 0.05). The number of covariates was selected based on the events‐per‐variable criterion (≥ 10 events per variable). This was done to reduce the risk of overfitting. Variables were assessed for multicollinearity (variance inflation factor [VIF] < 3.0; Supporting Table 1) and retained in the final model if they satisfied the proportional hazards assumption, which was formally evaluated using time‐dependent covariates.

## 3. Results

### 3.1. Baseline Characteristics and mNUTRIC Score by Survival Outcomes of the Patients

This study enrolled a total of 355 patients (Table 1). The median age was found to be 72 years (interquartile range, IQR, 62.0–79.0), and 218 patients were male (61.4%). Of the total cohort, 124 patients (34.9%) were diagnosed with sepsis and 231 patients (65.1%) with septic shock. Within 30 days, the in‐hospital mortality rates were 31.5% (*n* = 39) in the sepsis group and 29.9% (*n* = 69) in the septic shock group.

**TABLE 1:** Baseline characteristics and mNUTRIC scores according to 30‐day survival status.

Characteristics	Total (*N* = 355)	Survivors (*n* = 247)	Nonsurvivors (*n* = 108)	*p*
	*n* (%) or median [IQR]	*n* (%) or median [IQR]	*n* (%) or median [IQR]	
Age, years	72.0 [62.0–79.0]	72.0 [62.0–79.0]	71.5 [62.3–79.0]	0.809
Sex, male	218 (61.4)	146 (59.1)	72 (66.7)	0.178
Alcohol consumption, yes	48 (13.5)	39 (15.8)	9 (8.3)	0.059
Smoking, yes	38 (10.7)	26 (10.5)	12 (11.1)	0.870
Weight decreased, yes	54 (15.2)	36 (14.6)	18 (16.7)	0.614
GI dysfunction, yes	39 (11.0)	23 (9.3)	16 (14.8)	0.127
Comorbidities^†^				
Cancer, yes	121 (34.1)	86 (34.8)	35 (32.4)	0.659
Immunosuppression, yes	75 (21.1)	41 (16.6)	34 (31.5)	0.002
CKD, yes	39 (11.0)	24 (9.7)	15 (13.9)	0.247
BMI, kg/m^2^	21.8 [19.5–24.7]	21.6 [19.4–24.4]	22.5 [19.8–24.8]	0.123
Septic shock, yes	231 (65.1)	162 (65.6)	69 (63.9)	0.757
MAP < 65 mmHg, Day 1	265 (74.6)	183 (74.1)	82 (75.9)	0.714
Lactate ≥ 2 mmol/L, Day 1	291 (82.0)	204 (82.6)	87 (80.6)	0.539
SOFA score, points	11 [9–13]	11 [9–12]	12 [10–13]	0.002
APACHE II score, point	30.0 [25.0–34.0]	29.0 [25.0–34.0]	31.5 [27.3–35.0]	0.016
Source of infection				< 0.001
Pulmonary	196 (55.2)	123 (49.8)	73 (67.6)	
Intraabdominal	52 (14.6)	41 (16.6)	11 (10.2)	
Urinary tract	44 (12.4)	42 (17.0)	2 (1.9)	
Multiple	20 (5.6)	13 (5.3)	7 (6.5)	
Others	43 (12.1)	28 (11.3)	15 (13.9)	
mNUTRIC score, points	6 [5–7]	6 [5–7]	6 [5–7]	0.024
Low‐risk group (0–5)	138 (38.9)	108 (43.7)	30 (27.8)	0.005
High‐risk group (6–9)	217 (61.1)	139 (56.3)	78 (72.2)	

*Note*. APACHE II, Acute Physiology and Chronic Health Evaluation II; mNUTRIC, modified Nutrition Risk in the Critically Ill.

Abbreviations: BMI, body mass index; CKD, chronic kidney disease; GI, gastrointestinal; ICU, intensive care unit; MAP, mean arterial pressure; SOFA, sequential organ failure assessment.

^†^Multiple responses.

The median mNUTRIC score was 6 in both groups, the overall distribution of mNUTRIC scores was significantly different between survivors and nonsurvivors (*p* = 0.024). Overall, 217 patients (61.1%) were classified as high risk (≥ 6 points), with a significantly higher proportion in the nonsurvival group than in the survival group (72.2% vs. 56.3%, *p* = 0.005). The frequency distribution of mNUTRIC scores by 30‐day in‐hospital mortality status is shown in Supporting Figure 1.

The mortality group had significantly greater levels of immunosuppression (*p* = 0.002), SOFA score (*p* = 0.002), and APACHE II score (*p* = 0.016) than the survival group. In addition, the distribution of infection sources differed significantly between the two groups (*p* < 0.001) (Table 1).

### 3.2. Nutritional Status and Clinical Characteristics During ICU Stay by 30‐Day Survival Status of the Patients

The 30‐day nonsurvival group demonstrated significantly lower average energy intake (kcal/day, *p* = 0.049), lower energy intake adequacy during the late phase (Days 4–7) (*p* < 0.001), and lower protein intake adequacy during the late phase (Days 4–7) (*p* = 0.005) compared with the survival group (Table 2).

**TABLE 2:** Nutritional status and clinical characteristics during ICU stay according to 30‐day survival status.

Characteristics	Total (*N* = 355)	Survivors (*n* = 247)	Nonsurvivors (*n* = 108)	*p*
	*n* (%) or median [IQR]	*n* (%) or median [IQR]	*n* (%) or median [IQR]	
CRRT, yes	133 (37.5)	72 (29.1)	61 (56.5)	< 0.001
Mechanical ventilation, yes	304 (85.6)	204 (82.6)	100 (92.6)	0.013
Vasopressor use, yes	325 (91.5)	220 (89.1)	105 (97.2)	0.011
MAP < 65 mmHg, Day 7	183 (51.5)	118 (47.8)	65 (60.2)	0.031
Lactate ≥ 2 mmol/L, Day 7	171 (48.2)	93 (37.7)	78 (72.2)	< 0.001
Nutritional parameters				
Early enteral nutrition (< 48 h), yes	158 (44.5)	108 (43.7)	50 (46.3)	0.654
Average energy intake, kcal/day	582.3 [403.5–842.6]	594.9 [423.8–878.1]	507.0 [348.6–782.8]	0.049
Adequacy of energy intake (Days 1–3), %	22.6 [10.8–41.4]	24.5 [10.7–44.0]	18.8 [11.1–36.5]	0.156
Adequacy of energy intake (Days 4–7), %	56.2 [39.0–76.5]	59.1 [40.3–81.4]	49.3 [34.5–66.8]	< 0.001
Nonnutritional energy, % of total energy^†^	33.3 [20.0–50.0]	33.3 [19.3–50.0]	34.7 [20.2–55.6]	0.522
Average protein intake, g/day	27.1 [15.4–41.7]	27.7 [16.1–42.5]	23.9 [13.9–41.0]	0.125
Adequacy of protein intake (Days 1–3), %	15.4 [0.0–34.4]	16.8 [0.0–36.5]	13.0 [0.0–29.1]	0.421
Adequacy of protein intake (Days 4–7), %	54.1 [33.8–79.5]	55.8 [36.3–85.0]	47.0 [26.1–72.1]	0.005
Nutrition delivery route				0.002
EN only	242 (68.2)	181 (73.3)	61 (56.5)	
EN + PN	113 (31.8)	66 (26.7)	47 (43.5)	
Energy intake, EN only (*n* = 242)	450.0 [263.3–771.4]	485.7 [285.7–801.8]	385.7 [221.4–714.3]	0.069
Energy intake, EN + PN (*n* = 113)	585.0 [407.3–867.7]	663.7 [453.2–952.6]	488.5 [300.0–697.7]	0.004
Gastrointestinal complications, yes	172 (48.5)	118 (47.8)	54 (50.0)	0.699
Refeeding syndrome, yes	149 (42.0)	108 (43.7)	41 (38.0)	0.312
Length of ICU stay, days	12 [8–19]	12 [7–21]	12 [8–16]	0.473

Abbreviations: EN, enteral nutrition; CRRT, continuous renal replacement therapy; ICU, intensive care unit; MAP, mean arterial pressure; PN, parenteral nutrition.

^†^Analysis restricted to patients who received any propofol and/or dextrose water (*n* = 271).

The combined EN + PN group was more prevalent among those who did not survive within 30 days (*p* = 0.002). Within this subgroup, energy intake was significantly lower among those who did not survive within 30 days (*p* = 0.004). Patients in the 30‐day nonsurvival group more frequently required CRRT (*p* < 0.001), mechanical ventilation (*p* = 0.013), and vasopressor support (*p* = 0.011). They also exhibited a higher proportion of MAP < 65 mmHg (*p* = 0.031) and blood lactate levels ≥ 2 mmol/L (*p* < 0.001) on Day 7 after admission.

### 3.3. Nutritional Risk Stratification Using the mNUTRIC Score and 30‐Day Cumulative Survival

ROC analysis was performed to determine an appropriate threshold for assessing patients at high nutritional risk and to assess the prognostic performance of the mNUTRIC score in predicting mortality within 30 days of hospital admission. The analysis revealed an AUC of 0.579 (95% CI: 0.517–0.642). ROC analysis was conducted to assess the prognostic performance of the mNUTRIC score for 30‐day in‐hospital mortality, yielding an AUC of 0.579 (95% CI: 0.517–0.642). The Youden index identified 5.5 as the statistically derived cutoff value. Performance metrics for thresholds of 5 and 6 points were further compared to assess clinical applicability: The 5‐point threshold demonstrated higher sensitivity (88.0%) but lower specificity (19.8%) (Youden index = 0.08), whereas the 6‐point threshold showed improved specificity (43.7%) with acceptable sensitivity (72.2%) (Youden index = 0.16). The comparative assessment was used to select a score ≥ 6 as an exploratory high‐risk stratification threshold for this study.

**FIGURE 2:**
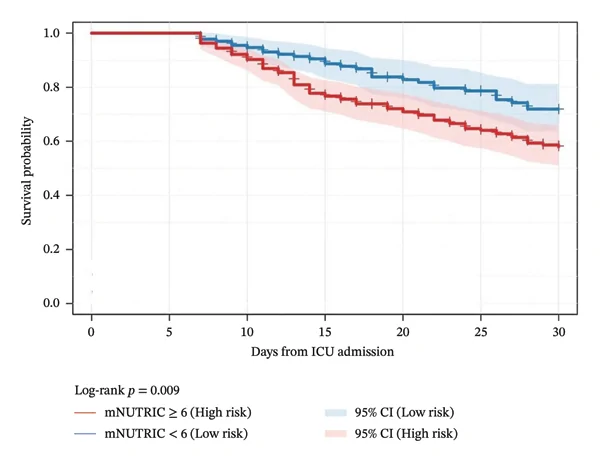
Kaplan–Meier curves for 30‐day survival in patients with sepsis and septic shock, stratified by mNUTRIC.

A Kaplan–Meier survival analysis was performed to compare the 30‐day cumulative survival rates of the low‐risk group (0–5 points) and the high‐risk group (6–9 points), as determined by the mNUTRIC score (Figure 2). The mean survival time was 26.59 days (95% CI: 25.411–27.767) in the low‐risk group (*n* = 138) and 24.22 days (95% CI: 23.089–25.351) in the high‐risk group (*n* = 217). The log‐rank test showed a statistically significant survival difference between the two groups (*p* = 0.009) (Figure 2).

### 3.4. Impact of the Patient’s Nutritional Status and Clinical Characteristics on 30‐Day In‐Hospital Mortality

In the multivariable model after adjustment for confounding factors (Table 3), a 10% increase in protein adequacy between Days 4 and 7 was linked to a lower risk of in‐hospital mortality within 30 days (HR = 0.906, 95% CI: 0.824–0.997, *p* = 0.043). Similarly, an mNUTRIC score of at least 6 was also related to an increased risk of in‐hospital mortality within 30 days (HR = 1.685, 95% CI: 1.020–2.781, *p* = 0.041).

**TABLE 3:** Cox proportional hazards regression analysis for 30‐day in‐hospital mortality.

Predictors	Unadjusted HR (95% CI)	*p*	Adjusted HR^†^ (95% CI)	*p*
Nutritional status				
mNUTRIC ≥ 6 (high risk)	1.729 (1.135–2.635)	0.011	1.685 (1.020–2.781)	0.041
Adequacy of energy intake (Days 4–7), per 10% point	0.868 (0.802–0.938)	< 0.001	0.952 (0.849–1.068)	0.400
Adequacy of protein intake (Days 4–7), per 10% point	0.910 (0.856–0.968)	0.003	0.906 (0.824–0.997)	0.043
Nutrition delivery route: EN + PN	1.600 (1.094–2.341)	0.016	1.421 (0.957–2.111)	0.082
General characteristics				
Sex, male	1.378 (0.923–2.056)	0.117	1.278 (0.847–1.930)	0.243
Immunosuppression, yes	1.680 (1.119–2.521)	0.012	1.366 (0.880–2.119)	0.164
Clinical characteristics				
SOFA score, per 1 point	1.086 (1.019–1.158)	0.012	0.960 (0.887–1.040)	0.318
CRRT, yes	2.370 (1.620–3.468)	< 0.001	1.882 (1.242–2.851)	0.003
MAP < 65 mmHg, Day 7	1.501 (1.021–2.206)	0.039	1.500 (1.005–2.239)	0.047
Lactate ≥ 2 mmol/L, Day 7	3.048 (2.015–4.609)	< 0.001	2.778 (1.817–4.247)	< 0.001

Abbreviations: CI, confidence interval; CRRT, continuous renal replacement therapy; EN, enteral nutrition; HR, hazard ratio; MAP, mean arterial pressure; mNUTRIC, modified Nutrition Risk in the Critically Ill; PN, parenteral nutrition; SOFA, sequential organ failure assessment.

^†^Adjusted HRs were estimated using a multivariable Cox proportional hazards model including all variables presented in the table. Reference categories were low mNUTRIC risk, EN only, female sex, no immunosuppression, no CRRT, MAP ≥ 65 mmHg on Day 7, and lactate < 2 mmol/L on Day 7. The model included 355 patients and 108 mortality events.

Among clinical variables, blood lactate ≥ 2 mmol/L on Day 7 was related to a higher risk of 30‐day in‐hospital mortality (HR = 2.778, 95% CI: 1.817–4.247, *p* < 0.001), as was the use of CRRT (HR = 1.882, 95% CI: 1.242–2.851, *p* = 0.003). A MAP < 65 mmHg on Day 7 was also linked to increased risk of in‐hospital mortality within 30 days (HR = 1.500, 95% CI: 1.005–2.239, *p* = 0.047).

As an exploratory sensitivity analysis, the adequacy of late‐phase protein during ICU stays from Day 4 to Day 7 was categorized as < 50%, 50%–79%, or ≥ 80%. We performed Cox regression using the same covariates as the primary model, with energy adequacy excluded to avoid overlap with protein adequacy. Compared with the < 50% group, the ≥ 80% group had a lower adjusted hazard of 30‐day in‐hospital mortality (HR = 0.496, 95% CI: 0.293–0.840, *p* = 0.009) (Supporting Table 2).

## 4. Discussion

In this retrospective analysis of 355 adults who survived the initial 7 days and received EN for sepsis or septic shock, greater protein adequacy during the late acute phase (Days 4–7) was independently associated with lower rates of in‐hospital mortality within 30 days, alongside baseline mNUTRIC risk. This relationship remained significant after adjustment for key clinical confounders, underscoring the prognostic relevance of timely protein delivery during this phase. These findings suggest that phase‐specific nutritional assessment, when integrated with clinical markers of sepsis, may support timely multidisciplinary care and contribute to improved patient outcomes. Among the nutritional parameters examined, protein adequacy during the late phase (Days 4–7) was strongly related to 30‐day in‐hospital mortality. Each 10% increase in protein adequacy during this period corresponded to a reduction in mortality risk, with a particularly pronounced benefit observed when ≥ 80% of target requirements was achieved. Similar findings have been reported in prior studies of critically ill populations, including patients with sepsis, in which higher protein adequacy during the late phase was associated with improved survival [19, 29].

However, substantial heterogeneity exists across studies with respect to both the timing of nutritional assessment and the recommended protein targets. Across broader critically ill populations, achieving early nutritional targets has been linked to improved outcomes [30, 31]. In contrast, an early increase in protein provision has also been reported to elevate mortality risk [32]. Studies focusing specifically on patients with sepsis have also yielded inconsistent findings. Wen et al. [33] reported higher protein intake in survivors compared with nonsurvivors during Days 3–7 after admission. Conversely, de Koning et al. [34] reported that high‐protein feeding (≥ 1.2 g/kg/day) during Days 4–7 was connected with a higher mortality rate than a moderate protein intake (0.8–1.2 g/kg/day). Similarly, Park et al. [35] highlighted the complexity of this temporal relationship, reporting that low protein delivery (< 1.0 g/kg/day) on Day 7 of the postacute phase was strongly linked to a higher 28‐day mortality rate.

Accordingly, these findings highlight the complex and multifaceted nature of the timing and clinical efficacy of protein provision. This marked discrepancy may be attributed to variations in protein requirements and tolerance limits depending on the presence of acute kidney injury (AKI). AKI is a prevalent complication among patients suffering from sepsis and septic shock. Recent literature and clinical guidelines focusing on critically ill patients with AKI suggest that high protein intake under these conditions relates to adverse clinical outcomes. In particular, excessive protein provision increases the risk of mortality in the absence of CRRT [14, 36]. Therefore, when determining protein delivery, an individualized approach that comprehensively integrates both the patient’s renal function and the potential plan for ongoing CRRT may be clinically relevant.

Among the nutritional parameters examined, high mNUTRIC risk showed the strongest association with 30‐day in‐hospital mortality. Higher mNUTRIC scores reflect both greater nutritional vulnerability and more severe underlying illness [37]. Patients classified as high risk may be particularly vulnerable to delays or interruptions in nutritional support and to progressive organ dysfunction, which may collectively contribute to adverse outcomes.

Consistent with the present findings, previous meta‐analyses have reported robust associations between high mNUTRIC risk and increased short‐term mortality, including at 28 and 30 days and during hospital stay [25, 38, 39]. By integrating acute‐phase illness severity with nutritional status, the mNUTRIC framework may offer advantages over traditional nutritional screening tools, in critically ill populations, such as the Nutritional Risk Screening 2002 (NRS‐2002) and the Mini Nutritional Assessment–Short Form (MNA‐SF) [35]. In the present multivariable analysis, baseline SOFA score was no longer statistically significant, whereas high mNUTRIC risk (score ≥ 6) remained independently associated with 30‐day mortality. This finding may partly reflect the incorporation of SOFA within the mNUTRIC score, allowing mNUTRIC to capture baseline organ dysfunction together with age, comorbidity burden, and nutritional vulnerability. This interpretation is consistent with previous studies in critically ill cohorts showing that mNUTRIC may provide prognostic information beyond conventional acuity scores [38, 40].

However, in the current cohort, the mNUTRIC score showed limited discriminative performance, as reflected by the modest AUC and low specificity. Therefore, the mNUTRIC ≥ 6 threshold should be considered an exploratory risk‐stratification threshold rather than as a conclusive, standalone screening cutoff. Critical care nurses should interpret mNUTRIC results alongside the patient’s overall clinical status and other major clinical indicators, rather than relying solely on this screening tool.

In our cohort, neither early enteral nutrition (EEN) within 48 h nor nutritional adequacy during Days 1–3 was significantly associated with 30‐day mortality, which may be partly attributable to profound hemodynamic instability during the early acute phase. Because 97.2% of nonsurvivors and 89.1% of survivors required active vasopressor support, early feeding may have been ineffective, poorly tolerated, or clinically inappropriate during severe shock. The current literature supports deferring EN during unstable shock because of the risk of mesenteric ischemia and prioritizing macrovascular resuscitation before advancing nutritional delivery [14], which is consistent with systematic reviews reporting heterogeneous outcomes associated with EEN [12, 13]. Moreover, severe metabolic stress in sepsis is accompanied by substantial endogenous energy production [9]. In view of the link between enteral feeding intolerance and higher mortality rates among patients with septic shock [7], gradual advancement of nutritional delivery may represent a cautious strategy for balancing feeding tolerance against the risk of overfeeding [15]. Furthermore, late‐phase energy adequacy (Days 4–7) was no longer statistically significant in the final multivariable model, consistent with the inconsistent findings reported in previous studies [33, 41]. This finding may be related to impaired calorie utilization during acute sepsis [29].

Given the high‐acuity profile of the present cohort, including a median SOFA score of 11 and septic shock in 65.1% of patients, the transition toward metabolic recovery may have occurred beyond Day 7 in some patients. In addition, nonnutritional sources accounted for 33.3% of total caloric intake, consistent with international reports of 30.7%–36.1% [42]. Failure to account for these sources may increase the risk of overfeeding, whereas excessive caloric restriction because of concerns regarding propofol‐derived calories may contribute to protein underdelivery. Therefore, careful consideration of nonnutritional calories is essential when balancing energy provision with protein optimization in critically ill patients [43].

In the current study, the strongest clinical factor associated with 30‐day in‐hospital mortality, apart from nutritional status, was a blood lactate level ≥ 2 mmol/L on Day 7 after admission. Previous research has also indicated a potential link between thiamine deficiency and increased lactate levels [44]. However, thiamine levels could not be assessed using the electronic medical records available for this study. More studies are needed to understand the link between thiamine deficiency, lactate levels, and clinical outcomes in patients with sepsis or septic shock. Patients receiving CRRT also exhibited higher 30‐day in‐hospital mortality. This observation underscores the profound disease severity among patients with acute renal failure rather than a direct detrimental effect of CRRT itself, consistent with prior study [45]. From a nutritional perspective, patients undergoing CRRT may experience substantial losses of amino acids and protein, leading to increased protein requirements.

Finally, it was identified that a MAP < 65 mmHg on Day 7 of ICU admission was a factor significantly associated with mortality. Persistent hypotension at this stage may indicate incomplete hemodynamic stabilization following the acute phase of illness [46]. Reduced MAP may also reflect compromised gastrointestinal perfusion. Accordingly, when EN is administered in hemodynamically unstable patients, gradual advancement with close monitoring for gastrointestinal complications is recommended.

### 4.1. Limitations

This study has several limitations. First, the exclusion of patients who died early, were discharged within 7 days, transitioned to oral intake, or did not receive EN within 7 days may have introduced survivor bias and immortal time bias, because both nutritional adequacy during Days 4–7 and clinical indicators on Day 7 could only be assessed among patients who remained in the cohort. This restriction limited the analysis to patients who stabilized after the initial acute phase and may have excluded the sickest patients who died early. Therefore, the link between high nutritional risk and mortality may have been underestimated in the broader sepsis population. Second, the unusually high proportion of septic shock in our cohort (65.1%) may reflect selection bias related to the single tertiary referral setting and the minimum ICU stay criterion of 48 h. This limits the findings’ generalizability to patients with sepsis. Third, the use of electronic health records may have introduced missing data and measurement error. Fourth, residual time‐varying confounding may remain because nutritional intake and physiological status changed during the ICU course. Although the proportional hazards assumption was evaluated, daily changes in nutritional exposure and clinical status could not be fully modeled. Fifth, subgroup analyses stratified by the mNUTRIC risk group could not be performed; therefore, whether the link between protein adequacy and mortality differed according to baseline nutritional risk remains unclear. Sixth, the relationship between late‐phase protein adequacy and lower mortality was observed only in the 2020–2024 cohort and not in the 2015–2019 cohort (Supporting Table 3). This suggests the presence of possible period‐specific confounding factors. It is imperative that further prospective multicenter research is conducted in order to validate these findings. Finally, outcomes were restricted to mortality within 30 days of hospital admission. Due to the observational design, causal inferences cannot be established.

## 5. Conclusion

Inadequate protein intake and nutritional risk were found to be independently linked to 30‐day in‐hospital mortality among critically ill patients suffering from sepsis and septic shock who survived the initial 7 days and received EN. In particular, lower protein delivery during Days 4–7 and a higher mNUTRIC score remained independently associated with poorer outcomes after adjusting for clinical covariates. These findings suggest that continued monitoring of protein adequacy, combined with early nutritional risk assessment, may help identify patients at high risk of adverse clinical outcomes.

For nursing management, the importance of structured nutritional intake monitoring and standardized nutritional screening within multidisciplinary intensive care is understood by these findings. Further prospective multicenter studies are necessary to clarify the association of phase‐specific protein delivery and clinical instability indicators with mortality. These studies should also determine the optimal mNUTRIC cutoff.

## Author Contributions

Jeeyoung Park: conception and design of the study, acquisition of data, formal analysis, interpretation of data, writing–original draft, and writing–review and editing. JiYeon Choi: writing–original draft and writing–review and editing. Suk Jeong Lee: writing–original draft and writing–review and editing. Youn‐Jung Son: conception and design of the study, data analysis, interpretation of data, supervision, writing–original draft, and writing–review and editing.

## Funding

This work was supported by the National Research Foundation of Korea (NRF) grant funded by the Korean government (MSIT) (Grant No. RS‐2025–00562535).

## Ethics Statement

The study protocol was approved by the IRB of Seoul National University Hospital (IRB No. H‐2311‐090‐1484). The study was conducted in accordance with the Declaration of Helsinki. Owing to the retrospective design of the study and the use of anonymized electronic medical record data, the requirement for written informed consent was formally waived by the Institutional Review Board.

## Consent

Please see the Ethics Statement.

## Conflicts of Interest

The authors declare no conflicts of interest.

## Supporting Information

Additional supporting information can be found online in the Supporting Information section.

## Supporting information


**Supporting Information 1** Supporting Table 1: VIFs for covariates in the multivariable model. Supporting Table 2: Days 4–7 protein intake adequacy groups associated with 30‐day mortality in critically ill patients with sepsis and septic shock. Supporting Table 3: Stratified multivariate cox proportional hazards regression analysis for 30‐day in‐hospital mortality. Supporting Figure 1: Frequency distribution of mNUTRIC scores by 30‐day mortality status.

**Supporting Information 2** STROBE Statement—checklist of items that should be included in reports of observational studies.

## Data Availability

The datasets used and/or analyzed during the current study are available from the corresponding author on reasonable request.
