# Supplementary material for: Nutritional Risk and 30‐Day In‐Hospital Mortality in Adults With Sepsis and Septic Shock: A Retrospective Cohort Study
Source: J Nurs Manag. 2026 Sep 9;2026:3010166. doi: 10.1155/jonm/3010166 (PMC13559008; doi:10.1155/jonm/3010166)
Supplement: Supplementary file 1 [file JONM-2026-3010166-s001.docx]

STROBE Statement—checklist of items that should be included in reports of observational studies

| **Categories** | Item | Recommendation | | Response |
| --- | --- | --- | --- | --- |
| **Title and abstract** | 1 | (*a*) Indicate the study’s design with a commonly used term in the title or the abstract |  | The study design was identified as a retrospective cohort study in the title and abstract **(pp. 1and 4)**. |
|  |  | (*b*) Provide in the abstract an informative and balanced summary of what was done and what was found | | The abstract provided a balanced summary of the background, methods, study sample, principal findings, conclusions, and implications for nursing management. **(pp. 4–5)** |
| Introduction | | | |  |
| Background/rationale | 2 | Explain the scientific background and rationale for the investigation being reported | | The scientific background and rationale for examining nutritional status and mortality in patients with sepsis or septic shock were described in the Introduction. **(pp. 6–7)** |
| Objectives | 3 | State specific objectives, including any prespecified hypotheses | | The study objective and prespecified variables of interest were stated at the end of the Introduction. **(p. 7-8)** |
| Methods | | | |  |
| Study design | 4 | Present key elements of study design early in the paper | | The principal elements of the retrospective cohort design were presented at the beginning of the Methods section. **(p. 8)** |
| Setting | 5 | Describe the setting, locations, and relevant dates, including periods of recruitment, exposure, follow-up, and data collection | | The study setting, location, study period, exposure-assessment period, outcome period, and data sources were described in the Methods section. **(pp. 8–10)** |
| Participants | 6 | (***a*) *Cohort study***—Give the eligibility criteria, and the sources and methods of selection of participants. Describe methods of follow-up  *Case-control study*—Give the eligibility criteria, and the sources and methods of case ascertainment and control selection. Give the rationale for the choice of cases and controls  *Cross-sectional study*—Give the eligibility criteria, and the sources and methods of selection of participants | | Eligibility criteria and participant selection are described in Section 2.1 **(p. 8)**. The follow-up methods, including the censoring criteria for patients discharged alive within 30 days, are detailed in Section 2.5, Data Analysis **(p. 10)**. |
|  |  | (*b*) *Cohort study*—For matched studies, give matching criteria and number of exposed and unexposed  *Case-control study*—For matched studies, give matching criteria and the number of controls per case | | NA |
| Variables | 7 | Clearly define all outcomes, exposures, predictors, potential confounders, and effect modifiers. Give diagnostic criteria, if applicable | | The outcome, nutritional exposures, clinical predictors, potential confounders, and relevant clinical thresholds were defined in Sections 2.2 (Measures) and 2.3 (Outcome Variable) **(pp. 8–9)**. |
| Data sources/ measurement | 8* | For each variable of interest, give sources of data and details of methods of assessment (measurement). Describe comparability of assessment methods if there is more than one group | | Data sources and measurement methods were described in Sections 2.2 (Measures) and 2.4 (Data Collection) **(pp. 8–10)**. The same electronic medical record sources and definitions were applied to the survival groups. |
| Bias | 9 | Describe any efforts to address potential sources of bias | | Analytic efforts to reduce confounding and model misspecification included clinically informed covariate selection, an events-per-variable criterion, multicollinearity assessment, and proportional hazards assumption testing, as described in Section 2.5, Data Analysis **(pp. 10–11)**. |
| Study size | 10 | Explain how the study size was arrived at | | All eligible patients during the study period were included. The participant selection process and final study size are presented in Section 2.1 **(p. 8)** and Figure 1 **(p. 27)**. |
| Quantitative variables | 11 | Explain how quantitative variables were handled in the analyses. If applicable, describe which groupings were chosen and why | | Quantitative variables were handled as continuous measures or categorized using clinical guidelines and exploratory thresholds. The rationales for these groupings are detailed in Section 2.2, Measures **(pp. 8–9)**. |
| Statistical methods | 12 | (*a*) Describe all statistical methods, including those used to control for confounding | | Statistical methods, including group comparisons, ROC analysis, Kaplan–Meier analysis, Cox proportional hazards regression, confounder selection, overfitting control, multicollinearity assessment, and proportional hazards assumption testing, are described in Section 2.5, Data Analysis **(pp. 10–11)**. |
|  |  | (*b*) Describe any methods used to examine subgroups and interactions | | NA |
|  |  | (*c*) Explain how missing data were addressed | | Patients with incomplete or missing records were excluded during the initial participant selection process. Consequently, there were no missing data for the variables of interest in the final analytic cohort of 355 patients, and no statistical imputation methods were required, as shown in Figure 1 **(p. 27)**. |
|  |  | **(*d*) *Cohort study***—If applicable, explain how loss to follow-up was addressed  *Case-control study*—If applicable, explain how matching of cases and controls was addressed  *Cross-sectional study*—If applicable, describe analytical methods taking account of sampling strategy | | Follow-up and censoring procedures for the 30-day in-hospital mortality outcome are detailed in Section 2.5, Data Analysis **(p. 10)**. |
|  |  | (*e*) Describe any sensitivity analyses | | Exploratory sensitivity analyses regarding categorized protein adequacy and study-period stratification were conducted. The analyses and corresponding results are presented in Section 3.4 **(p. 13-14)** and Supplementary Tables 2 and 3. |
| Results | | | |  |
| Participants | 13* | (a) Report numbers of individuals at each stage of study—eg numbers potentially eligible, examined for eligibility, confirmed eligible, included in the study, completing follow-up, and analysed | | The number of individuals at each stage of the study, from initial screening to the final analytic cohort of 355 patients, is detailed in Figure 1 **(p. 27)**. |
|  |  | (b) Give reasons for non-participation at each stage | | Reasons for exclusion at each stage are specified in Figure 1 **(p. 27)**. |
|  |  | (c) Consider use of a flow diagram | | A flow diagram of the participant selection process is provided as Figure 1 **(p. 27)**. |
| Descriptive data | 14* | (a) Give characteristics of study participants (eg demographic, clinical, social) and information on exposures and potential confounders | | Participant demographic and clinical characteristics, nutritional exposures, and potential confounders were reported in the Results section **(pp. 11–12)** and Tables 1 and 2 **(pp. 29-30)**. |
|  |  | (b) Indicate number of participants with missing data for each variable of interest | | NA |
|  |  | (c) *Cohort study*—Summarise follow-up time (eg, average and total amount) | | All patients were followed for up to 30 days. The descriptive summary of the 30-day follow-up outcomes is presented in Sections 3.1 and 3.2 **(pp. 11–12)**. |
| Outcome data | 15* | ***Cohort study***—Report numbers of outcome events or summary measures over time | | The numbers of outcome events (30-day in-hospital mortality) and summary measures are reported in Sections 3.1 and 3.2 **(pp. 11–12)** and Tables 1 and 2 **(pp. 29–30)**. |
|  |  | *Case-control study—*Report numbers in each exposure category, or summary measures of exposure | |  |
|  |  | *Cross-sectional study—*Report numbers of outcome events or summary measures | |  |
| Main results | 16 | (*a*) Give unadjusted estimates and, if applicable, confounder-adjusted estimates and their precision (eg, 95% confidence interval). Make clear which confounders were adjusted for and why they were included | | Unadjusted and adjusted hazard ratios with 95% confidence intervals were reported in Table 3 **(p. 31)**. Covariate selection and the variables included in the multivariable model were described in Section 2.5, Data Analysis **(pp. 10–11)**, and in the footnote to Table 3 **(p. 31)**. |
|  |  | (*b*) Report category boundaries when continuous variables were categorized | | The category boundaries for categorized continuous variables (e.g., mNUTRIC score ≥ 6, MAP < 65 mmHg, lactate ≥ 2 mmol/L, and exploratory protein-adequacy groups) are explicitly reported in the Results section **(pp. 11–14)**, Tables 1–3 **(pp. 29-31)**, and Supplementary Table 2. |
|  |  | (*c*) If relevant, consider translating estimates of relative risk into absolute risk for a meaningful time period | | NA |
| Other analyses | 17 | Report other analyses done—eg analyses of subgroups and interactions, and sensitivity analyses | | Exploratory sensitivity analyses of categorized late-phase protein adequacy and study period were reported in Section 3.4 **(p. 13-4)** and Supplementary Tables 2 and 3. |
| Discussion | | | |  |
| Key results | 18 | Summarise key results with reference to study objectives | | The key findings were summarized in relation to the study objective at the beginning of the Discussion. **(p. 14)** |
| Limitations | 19 | Discuss limitations of the study, taking into account sources of potential bias or imprecision. Discuss both direction and magnitude of any potential bias | | Limitations were discussed, including survivor bias, immortal-time bias, selection bias, missing data, residual confounding, period-specific confounding, imprecision, and limited generalizability. **(pp. 18–19)** |
| Interpretation | 20 | Give a cautious overall interpretation of results considering objectives, limitations, multiplicity of analyses, results from similar studies, and other relevant evidence | | The findings were interpreted cautiously in relation to the study objective, previous evidence, the clinical context, and the study limitations. **(pp. 14–20)** |
| Generalisability | 21 | Discuss the generalisability (external validity) of the study results | | Generalizability was discussed in the Limitations section with reference to the restricted cohort and single tertiary-hospital setting. **(pp. 18–19)** |
| Other information | | | |  |
| Funding | 22 | Give the source of funding and the role of the funders for the present study and, if applicable, for the original study on which the present article is based | | Funding information and the funders' role are detailed in the Funding section. (p. 2) |

*NA: Not applicable.
