# Supplementary material for: Nutritional Risk and 30‐Day In‐Hospital Mortality in Adults With Sepsis and Septic Shock: A Retrospective Cohort Study
Source: J Nurs Manag. 2026 Sep 9;2026:3010166. doi: 10.1155/jonm/3010166 (PMC13559008; doi:10.1155/jonm/3010166)
Supplement: Supplementary file 2 [file JONM-2026-3010166-s002.docx]

**Supplementary Table 1.** VIFs for covariates in the multivariable model

| **Predictors** | **Non-standardized**  **coefficient** | | **Standardized coefficient** | ***t*** | ***p*** | **Statistics of collinearity** | |
| --- | --- | --- | --- | --- | --- | --- | --- |
|  | **B** | **standard**  **error** | ***β*** |  |  | **Tolerance** | **VIF** |
| (Constant) | 0.210 | 0.142 |  | 1.481 | 0.140 |  |  |
| mNUTRIC ≥ 6 (high risk) | 0.122 | 0.056 | 0.128 | 2.192 | 0.029 | 0.722 | 1.386 |
| Adequacy of energy intake  (days 4–7), per 10% point | -0.009 | 0.012 | -0.058 | -0.750 | 0.454 | 0.415 | 2.411 |
| Adequacy of protein intake  (days 4–7), per 10% point | -0.020 | 0.010 | -0.149 | -1.952 | 0.052 | 0.418 | 2.391 |
| Nutrition delivery route:  EN + PN | 0.087 | 0.051 | 0.088 | 1.694 | 0.091 | 0.915 | 1.093 |
| Sex, male | -0.042 | 0.048 | -0.044 | -0.863 | 0.389 | 0.960 | 1.041 |
| Immunosuppression, yes | 0.134 | 0.058 | 0.119 | 2.299 | 0.022 | 0.917 | 1.091 |
| SOFA score, per point | -0.012 | 0.010 | -0.079 | -1.281 | 0.201 | 0.652 | 1.534 |
| CRRT, yes | 0.173 | 0.052 | 0.180 | 3.334 | < 0.001 | 0.841 | 1.189 |
| Day 7 MAP < 65mmHg | -0.106 | 0.047 | -0.114 | -2.244 | 0.026 | 0.946 | 1.057 |
| Day 7 Lactate ≥ 2mmol/L | 0.222 | 0.047 | 0.239 | 4.699 | <0.001 | 0.949 | 1.054 |

***Note.*** CRRT, continuous renal replacement therapy; EN, enteral nutrition; MAP, mean arterial pressure; mNUTRIC, modified nutrition risk in the critically ill; PN, parenteral nutrition; SOFA, sequential organ failure assessment; VIF, variance inflation factor.

**Supplementary Table 2.** Day 4-7 protein intake adequacy groups associated with 30-day mortality in critically ill patients with sepsis and septic shock (*N* = 355)

| **Day 4-7 Protein adequacy** | **Mortality rate (%)** | **Unadjusted HR (95% CI)** | ***p*** | **Adjusted HR (95% CI)** † | ***p*** |
| --- | --- | --- | --- | --- | --- |
| < 50% | 53.7 | Reference | - | Reference | - |
| 50%-79% | 27.8 | 0.787 (0.506-1.223) | 0.287 | 0.639 (0.398-1.026) | 0.064 |
| ≥ 80% | 18.5 | 0.570 (0.343-0.948) | 0.030 | 0.496 (0.293-0.840) | 0.009 |

*Note.* CI, confidence interval; EN, enteral nutrition; HR, hazard ratio; mNUTRIC, modified nutrition risk in the critically ill; PN, parenteral nutrition; SOFA, sequential organ failure assessment. †Adjusted for sex, mNUTRIC ≥6 (high risk), route of nutritional support, Immunosuppression, SOFA score, continuous renal replacement therapy, Day 7 mean arterial pressure <65 mmHg, Day 7 Lactate ≥2 mmol/L

**Supplementary Table 3.** Stratified Multivariate Cox Proportional Hazards Regression Analysis for 30-Day In-Hospital Mortality

| **Predictors** | **2015–2019 Cohort (n=169)** | | **2020–2024 Cohort (n=186)** | |
| --- | --- | --- | --- | --- |
|  | **Adjusted HR**† **(95% CI)** | ***p*** | **Adjusted HR**† **(95% CI)** | ***p*** |
| *Nutritional status* |  |  |  |  |
| mNUTRIC ≥ 6 (high risk) | 1.489 (0.685–3.236) | 0.315 | 1.639 (0.780-3.443) | 0.192 |
| Adequacy of energy intake (days 4–7), per 10%p | 0.817 (0.662–1.009) | 0.060 | 1.049 (0.889-1.237) | 0.574 |
| Adequacy of protein intake (days 4–7), per 10%p | 0.998 (0.834–1.194) | 0.979 | 0.872 (0.769-0.990) | 0.035 |
| Nutrition delivery route: EN + PN† | 1.019 (0.555–1.872) | 0.951 | 2.286 (1.322-3.952) | 0.003 |
| *General characteristics* |  |  |  |  |
| Sex, male | 1.155 (0.623–2.141) | 0.647 | 0.663 (0.375-1.172) | 0.158 |
| Immunosupression, yes | 2.355 (1.212–4.576) | 0.011 | 0.840 (0.439-1.606) | 0.598 |
| *Clinical characteristics* |  |  |  |  |
| SOFA score, per 1point | 1.004 (0.890–1.132) | 0.951 | 0.939 (0.837-1.054) | 0.285 |
| CRRT, yes | 1.487 (0.786–2.812) | 0.223 | 2.517 (1.401-4.522) | 0.002 |
| Day 7 MAP < 65mmHg | 1.869 (0.988–3.537) | 0.055 | 1.053 (0.605-1.834) | 0.856 |
| Day 7 Lactate ≥ 2mmol/L | 3.080 (1.585–5.984) | <0.001 | 3.054 (1.691-5.516) | <0.001 |

***Note.*** CI, confidence interval; CRRT, continuous renal replacement therapy; EN, enteral nutrition; HR, hazard ratio; MAP, mean arterial pressure; mNUTRIC, modified nutrition risk in the critically ill; PN, parenteral nutrition; SOFA, sequential organ failure assessment. †Adjusted HRs were estimated using a multivariable Cox proportional hazards model including all variables presented in the table. Reference categories were low mNUTRIC risk, EN only, female sex, no immunosuppression, no CRRT, MAP ≥ 65 mmHg on Day 7, and lactate < 2 mmol/L on Day 7. The model included 355 patients and 108 mortality events.

**Supplementary Figure 1.** Frequency distribution of mNUTRIC scores by 30-day mortality status

***Note.*** mNUTRIC, modified Nutrition Risk in the Critically Ill
